# Serological Approaches for COVID-19: Epidemiologic Perspective on Surveillance and Control

**DOI:** 10.3389/fimmu.2020.00879

**Published:** 2020-04-24

**Authors:** Cheryl Yi-Pin Lee, Raymond T. P. Lin, Laurent Renia, Lisa F. P. Ng

**Affiliations:** ^1^Singapore Immunology Network, Agency for Science, Technology and Research (A^*^STAR), Singapore, Singapore; ^2^National Public Health Laboratory, National Centre for Infectious Diseases, Singapore, Singapore; ^3^Department of Microbiology and Immunology, Yong Loo Lin School of Medicine, National University of Singapore, Singapore, Singapore; ^4^Department of Biochemistry, Yong Loo Lin School of Medicine, National University of Singapore, Singapore, Singapore; ^5^Department of Infection and Microbiome, Institute of Infection, Veterinary and Ecological Sciences, University of Liverpool, Liverpool, United Kingdom

**Keywords:** SARS-CoV-2, COVID-19, detection, immunoassays, antibodies, spike, receptor binding domain, nucleocapsid

## Abstract

Since December 2019, the novel coronavirus, SARS-CoV-2, has garnered global attention due to its rapid transmission, which has infected more than two million people worldwide. Early detection of SARS-CoV-2 is one of the crucial interventions to control virus spread and dissemination. Molecular assays have been the gold standard to directly detect for the presence of viral genetic material in infected individuals. However, insufficient viral RNA at the point of detection may lead to false negative results. As such, it is important to also employ immune-based assays to determine one's exposure to SARS-CoV-2, as well as to assist in the surveillance of individuals with prior exposure to SARS-CoV-2. Within a span of 4 months, extensive studies have been done to develop serological systems to characterize the antibody profiles, as well as to identify and generate potentially neutralizing antibodies during SARS-CoV-2 infection. The vast diversity of novel findings has added value to coronavirus research, and a strategic consolidation is crucial to encompass the latest advances and developments. This review aims to provide a concise yet extensive collation of current immunoassays for SARS-CoV-2, while discussing the strengths, limitations and applications of antibody detection in SARS-CoV-2 research and control.

## Introduction

The ongoing pandemic, which originates from a newly emerged coronavirus, SARS-CoV-2, was discovered in the city of Wuhan in China's Hubei province in December 2019 (1). To date, due to rapid transmission globally, there are more than two million laboratory-confirmed human infection cases, with a few hundred thousand deaths across 210 countries and territories (https://www.who.int/emergencies/diseases/novel-coronavirus-2019/situation-reports/). This unprecedented crisis led to a worldwide effort to rapidly characterize the immunobiology of SARS-CoV-2, while mitigating further spread of this deadly pathogen.

**Figure 1:**
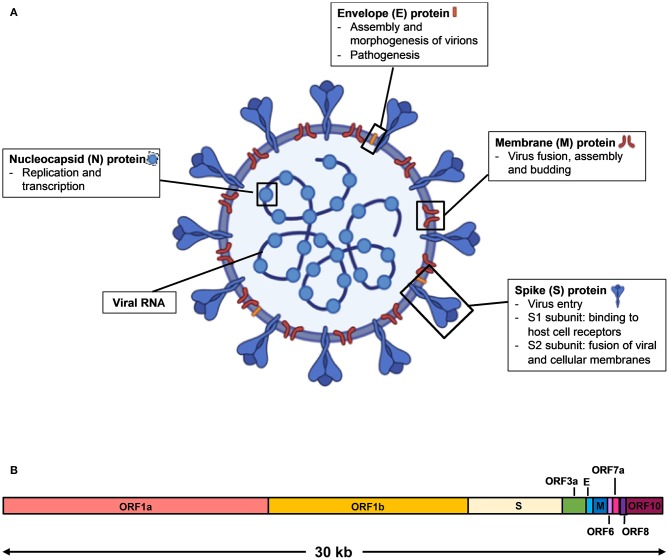
Schematic diagram of SARS-CoV-2 virus structure and genome organization. **(A)** The viral surface proteins, spike (S), envelope (E), and membrane (M) are embedded in a lipid bilayer. The single stranded positive-sense viral RNA is associated with the nucleocapsid (N) protein. Diagram was created with BioRender. **(B)** The genome organization of SARS-CoV-2 viral RNA, which is adapted from GenBank accession number: MN908947, is characterized by sequence alignment against two representative members of the *betacoronavirus* genus. The entire genome sequence is ~30 kilobases (kb) long.

SARS-CoV-2 is a single stranded, positive sense RNA virus that belongs to the *Coronaviridae* family of the *betacoronavirus* genus (2). It has a genome size of ~30 kilobases that encodes for multiple structural proteins comprising the spike (S), the envelope (E), the membrane (M), and the nucleocapsid (N), as well as non-structural proteins (3) (Figure 1). Infection by SARS-CoV-2 causes an acute respiratory disease termed the Coronavirus Disease 2019 (COVID-19). The clinical manifestations of COVID-19 form a spectrum, from being asymptomatic to fever with mild respiratory illness, to acute respiratory distress syndrome, and death from respiratory failure or associated complications (3–5). As the reported incubation period varies among different patient cohorts, it is often difficult to ascertain the actual day of onset, and infected subjects who are asymptomatic or pre-symptomatic may go undetected (5–7).

**Figure 2:**
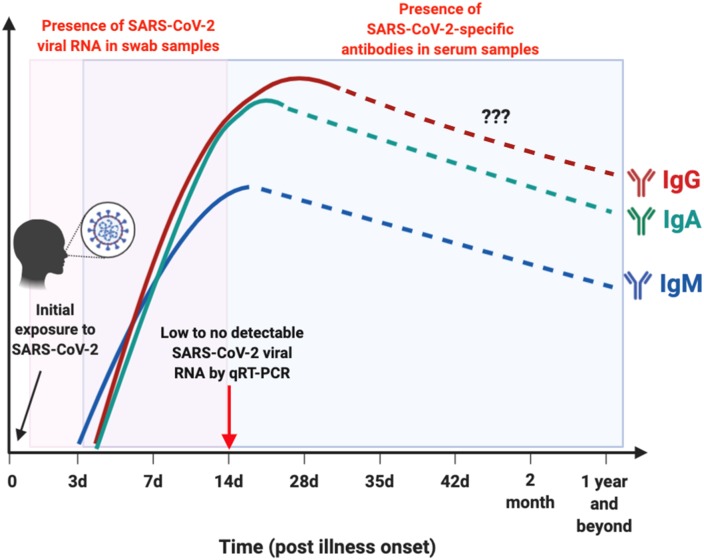
Schematic illustration on the window period of detection for either viral RNA or antibodies in SARS-CoV-2-infected individuals. Presence of SARS-CoV-2 viral RNA (boxed in pink) in throat or nasal swab of patients are typically undetectable by 14 day post illness onset (pio) (8, 9). SARS-CoV-2-specific antibodies (boxed in blue): IgM is detectable as early as 3 days pio, and peaks between 2 and 3 weeks pio (10, 11). IgM response was still detectable after more than 1 month pio (12). Both IgA and IgG are present as early as 4 days pio, and peaks after 2 weeks pio in serum samples (10, 11, 13, 14). There are currently no reports on the presence of these SARS-CoV-2-specific antibodies in the later phase pio, as indicated by dotted lines. This depicts the importance of serological studies to identify individuals with current or prior exposure to SARS-CoV-2 that went undetected, by testing for either IgM, IgG, or IgA antibodies against SARS-CoV-2. Illustration was created using BioRender.

Early detection of SARS-CoV-2 infection is one of the crucial interventions to control virus transmission. With the discovery of the virus, numerous diagnostic assays using quantitative reverse transcriptase PCR (qRT-PCR) were developed (3). qRT-PCR is the reference standard for diagnosing infections with high sensitivity and accuracy in the Acute phase of illness. SARS-CoV-2 viral RNA has been detected in both throat and nasal swabs of infected individuals by qRT-PCR, which becomes almost undetectable by 14 days post-illness onset (pio) (or symptom onset) (8, 9) (Figure 2). Apart from being costly and time consuming to perform, false negative results may arise due to improper handling of nucleic acid samples, inadequate and variable sampling resulting in insufficient viral genetic material at the point of detection (after 14 days pio), or biological variation on when viral RNA is detectable by qRT-PCR (10, 15). With the limitations of qRT-PCR, immunoassays may offer another avenue to reduce undiagnosed cases, with the advantage that rapid test formats may deliver results in a relatively shorter time and lower cost (10).

## Current Immune-Based Detection Approaches Against SARS-CoV-2

Immunoassays are another diagnostic approach that can provide information on both active viral infections and past exposures (Figure 2). To date, many commercial companies and research institutes have developed serological assays to detect SARS-CoV-2 antibodies from patient serum or plasma samples (16, 17). Closely related to another pathogen, Severe Acute Respiratory Syndrome coronavirus (SARS-CoV), these assays mainly target immunogenic coronavirus proteins: S protein, which is the most exposed viral protein, and N protein, which is abundantly expressed during infection (3, 14, 18). In addition, the receptor-binding domain (RBD), which is located along the S protein, is also a target of interest to detect the presence of SARS-CoV-2-specific antibodies (19, 20).

### Antibody Profiling of COVID-19 Patients

In recent pre-prints deposited in MedXriv and BioXriv, it was shown that both anti-SARS-CoV-2-IgM and IgG levels increase gradually along with infection phases, with IgM being detected as early as 3 days pio, which peaks between two to three weeks pio (10, 11). One study has reported that SARS-CoV-2-specific IgM is still present in the serum after 1 month pio (12). SARS-CoV-2-specific IgG antibodies, on the other hand, can be present as early as 4 days pio, and peak after 17 days pio (10, 11) (Figure 2). These observations are similar to what was previously reported during a SARS-CoV infection (21). However, interestingly, one study demonstrated that longitudinal profiling of both antibodies in a population of 63 COVID-19 patients showed no specific chronological order in terms of IgM and IgG seroconversion (10), which was also observed in patients infected with SARS-CoV and another human coronavirus, Middle East Respiratory Syndrome coronavirus (MERS-CoV) (22, 23). In addition, there seems to be no correlation between seroconversion rates with age, gender or time of hospitalization (10). These findings on SARS-CoV-2-specific antibodies seroconversion against the S viral protein suggest the importance to test for both IgM and IgG antibodies to confirm a positive infection.

Expectedly, similar to what was reported for SARS-CoV and MERS-CoV, both IgM and IgG levels seems to be correlated with disease severity, with a higher level of both antibodies present in patients with more severe SARS-CoV-2 infection (10, 11, 14, 24–26). In contrast to other flu-like infections such as influenza, instead of IgG1, IgG3 appears to be the dominant IgG subtype during SARS-CoV-2 infection (13, 27, 28).

### Specificity and Sensitivity of Immunoassays Against SARS-CoV-2

As a majority of the human population has prior exposure to endemic human coronavirus infections including alphacoronaviruses (229E and NL63), and other betacoronaviruses (OC42 and HKU1) (29), it is crucial to validate the specificity and sensitivity of current immunoassays against SARS-CoV-2 to avoid false positive outcomes. Within the S protein antigen, cross-reactivity was observed when samples were tested against SARS-CoV S and S1 subunit proteins, and to a smaller extent, with MERS-CoV S protein (Table 1). Interestingly, there was no cross-reactivity with the S1 subunit of MERS-CoV (14). The high level of cross-reactivity between SARS-CoV and SARS-CoV-2 can be attributed to the high degree of genetic homology (3, 14, 19). Furthermore, detailed analysis revealed a highly conserved S2 subunit domain across coronaviruses, which may explain for the cross-reactivity observed with only the S protein of MERS-CoV, and not with the S1 subunit (14, 19). These data suggest that using an S1 subunit-based immunoassay may be more specific than the entire S antigen for diagnosing SARS-CoV-2 infections.

**Table 1:** Immune-based assays developed against different SARS-CoV-2 viral proteins.

**Antigen**		**Antibody**	**Sample type**	**Specificity**	**References**
Spike (S)	Entire S	IgM, IgG	Patient serum	Not reported	(10, 11)
		IgG	Patient serum	Cross-react with SARS-CoV and MERS-CoV	(14)
		Not indicated	Patient plasma	Cross-react with SARS-CoV	(19)
		IgM, IgG, IgA	Patient serum or plasma	Not reported	(13)
	S1 subunit	IgG, IgA	Patient serum	Cross-react with SARS-CoV only	(14)
	S2 subunit	Not indicated	Patient plasma	Not reported	(19)
Receptor-binding domain (RBD)		IgG	Patient serum	Cross-react with SARS-CoV only	(14)
		Not indicated	Patient plasma	Cross-react with SARS-CoV	(19)
		IgG	Mouse serum	SARS-CoV RBD-induced antibodies cross-react to SARS-CoV-2 RBD	(20)
		IgM, IgG, IgA	Patient serum or plasma	Not reported	(13)
Nucleocapsid (N)		IgG	Patient serum	Cross-react with SARS-CoV only	(14)

Another immunogenic target, the RBD, which lies along the S protein is usually the target of many neutralizing antibodies against SARS-CoV (30). A substantial level of cross-reactivity by SARS-CoV RBD-induced antibodies to SARS-CoV-2 RBD was described (Table 1) (20). Of clinical relevance, these antibodies were also able to cross-neutralize SARS-CoV-2 pseudovirus infection, signifying the potential of an immunotherapy-based treatment (20). While one non-peer reviewed study has shown that RBD-based serological assays are more sensitive than S1 subunit-based assays in identifying antibodies in mild COVID-19 patients (14), other non-peer-reviewed studies have described a lower degree of antibody response to the RBD as compared to full-length S protein, plausibly reflecting the larger number of epitopes present on the larger S antigen (13, 19).

Due to a high level of similarity of 90% between SARS-CoV and SARS-CoV-2 N proteins, the N antigen of SARS-CoV was also used for serological detection of SARS-CoV-2-specific antibodies (Table 1) (14). These N-based assays were reported to be more sensitive than S1 subunit-based tests (14). The use of SARS-CoV antigens to diagnose SARS-CoV-2 infections may be reliable, given that SARS-CoV has not circulated in the human population since 2004 (3). In addition, an earlier report has demonstrated waning of SARS-CoV-specific antibodies, therefore being undetectable in 91% of patient serum samples after 6 years (31).

Since respiratory diseases are the hallmark of coronavirus infections, which activate mucosal immunity, several studies have exploited the detection of IgA to diagnose SARS-CoV-2 infection in patients (Table 1) (13, 14). Although a strong IgA response was also detected in COVID-19 patients where peak seroconversion was achieved by two weeks pio (Figure 2), IgA-based immunoassay has been hypothesized to be less specific than IgG-based ELISA due to cross-reactivity with serum samples from patients infected by other coronaviruses (14).

With the availability of immunoassays utilizing various coronavirus structural proteins, the use of more than one different antigen-based serological approach may be essential to establish a true positive SARS-CoV-2 infection. In addition, the use of saliva samples and other bodily fluid swabs as a less invasive alternative, which have been done for other viral infections including HIV and measles, should also be explored for serological testing of SARS-CoV-2 infections (32, 33).

### Identification of B-Cell Epitopes Against SARS-CoV-2 on Immunogenic Proteins

Apart from using immunoassays for the early detection of SARS-CoV-2 infected individuals, it is also critical to determine the regions where SARS-CoV-2-specific antibodies bind to help guide vaccine designs. Using SARS-CoV-derived B-cell epitopes that have been experimentally identified from positive B-cell assays (34), 49 out of 298 linear B-cell epitopes have an identical match with SARS-CoV-2 protein sequences without any mutations (3). Notably, majority of these matches were located at both the S and N viral antigens, with only 4 from the M protein, and none in the E protein (3). On the other hand, 6 conformational B-cell epitopes identified from the same database were located on the S antigen. However, unlike the linear epitopes, none of these mapped identically to the SARS-CoV-2 protein (3).

Further mapping the residues of linear B-cell epitopes onto available SARS-CoV S protein structure revealed several regions on the S2 subunit that may allow cross-neutralization of both SARS-CoV and SARS-CoV-2 (3, 35). In contrast, conformational B-cell epitopes mapped onto the S1 subunit, resulting in very few identical residues within SARS-CoV and SARS-CoV-2 (3). These findings indicate that SARS-CoV-specific antibodies targeting these discontinuous regions may not be able to cross-react with SARS-CoV-2 (3, 36). As these regions are computationally predicted, serological studies using patient samples are necessary to validate the importance of these regions for serology and in controlling SARS-CoV-2 infection. It also remains imperative to identify other SARS-CoV antibodies that may recognize the conformational epitopes of SARS-CoV-2 S protein, which can greatly reduce the amount of time needed to develop novel neutralizing antibodies.

## Applications of Immunoassays to Control SARS-CoV-2 Transmission

The findings derived from serological assays can provide valuable information that would help to support the diagnosis, treatment, and prevention of SARS-CoV-2 infections. Characterization of antibody profiles suggested that any suspected individuals with undetectable antibody levels against SARS-CoV-2 after 20 days pio may be a true negative case, since both anti-SARS-CoV-2 IgM or IgG seroconversion should have already occurred (10, 11). However, these findings may be limited to the relatively small sample size (<300 patients) and may require further validation with a larger cohort. In order to reinforce diagnosis, it would be advisable to perform multiple assays against different viral antigen.

In addition, the information of antibody seroconversion is crucial in determining the optimal timepoints to collect serum or plasma samples for immunoassay screening, as well as obtaining peripheral blood B cells for the generation of therapeutic monoclonal antibodies (37). Currently, in order to rapidly generate neutralizing monoclonal antibodies against SARS-CoV-2, repurposing of existing SARS-CoV-specific antibodies was demonstrated. To date, two human SARS-CoV-specific antibodies, CR3022 and 47D11, have been shown to recognize SARS-CoV-2 (38, 39). CR3022 recognizes an epitope along the RBD of SARS-CoV-2, which differs largely at the C-terminus residues to the RBD of SARS-CoV (38). Unfortunately, this variation in sequence impacted the ability of CR3022 to cross-neutralize SARS-CoV-2. Monoclonal antibody 47D11, on the other hand, targets the RBD along the S1 subunit of both SARS-CoV and SARS-CoV-2 with similar affinities, thereby enabling cross-neutralization against SARS-CoV-2 infection (39). While combinatory therapy has exhibited a stronger neutralization capability against SARS-CoV infection (40), a cocktail antibody approach for SARS-CoV-2 could be explored.

Surprisingly, reports on antibodies against the coronavirus E protein are scarce, possibly due to it being the smallest protein. However, the E antigen is involved in viral assembly, release of virions, as well as virus pathogenesis (41). It was demonstrated that recombinant coronaviruses lacking the E protein displayed significantly reduced viral titers, impaired viral maturation and produced avirulent virus progenies, suggesting a similar importance of E protein during SARS-CoV-2 infection (42, 43). Thus, it would be worthwhile to identify or generate neutralizing antibodies that are specific against the viral E protein.

During the course of an epidemic, one of the main challenges is the identification of asymptomatic infection. Since these individuals do not present any distinguishable symptoms, they could be the major source of transmission (10). Immunoassays may be able to detect mildly infected cases (14), which is important to ascertain the extent of community spread.

## Drawbacks of Serological Studies

While it is fast, robust and easy to perform, there are several limitations to serological assays. One of the major setbacks of immunoassays is the inability to detect the presence of infection during the early stage of disease, as antibodies take several days to be generated after exposure to foreign material (44). As such, a recent infection may provide false negative results during serological testing. Thus, the use of RT-PCR may be more suitable to diagnose an early acute SARS-CoV-2 infection. Furthermore, due to the unique genetic makeup of each individual, there would be an inherent variability of the antibody response (45). This could possibly explain the difference in antibody profiles elicited among individuals infected with SARS-CoV-2 (10).

Cross-reactivity could potentially be a limitation of immunoassays as it severely impacts the specificity and sensitivity of the test. Although the phylogenetically closest coronavirus, SARS-CoV, has not been reported to be circulating in the human population since 2004 (3), other endemic human coronaviruses may still pose a problem to accurately diagnose patients with true SARS-CoV-2 infection. While a recent study has demonstrated negligible cross-reactivity from human coronavirus, NL63, to SARS-CoV-2 (13), validation with other human coronaviruses remains to be investigated. In addition, prior findings on the S protein sequence and neutralization antigenicity of other coronaviruses suggest that antibodies neutralizing clinical human coronavirus isolates may not have the same degree of cross-reactivity with laboratory strains of human coronaviruses, thereby affecting the sensitivity of immunoassays (46–48).

## The Way Forward

Given the rapid increase in the number of confirmed COVID-19 cases coupled with the shortage in test kits to meet rising demands, decentralized point-of-care tests (POCT) may be another alternative to facilitate SARS-CoV-2 diagnosis. Such tests include lateral flow assay (LFA), which is a paper-based platform for the detection and quantification of analytes in complex mixtures (49). To design LFA for SARS-CoV-2 detection, an antibody specific to the viral antigen, or a viral antigen that is detectable by patient serum or plasma samples can be immobilized on a nitrocellulose membrane. Detection of binding between the analyte and capture antibody by a detector antibody will give rise to a colored line, closely resembling home pregnancy kits (50). POCT is advantageous as it is usually designed to be rapid, sensitive, highly accessible, and easily performed, requiring only a small amount of sample (50). Meanwhile, several hundreds of candidate POCTs are being evaluated for their applicability toward identifying SARS-CoV-2-infected individuals (50). However, POCTs can't replace RT-PCR and it is crucial that these developing tests are rigorously assessed prior to use. It is important to note that wrong use and interpretation could lead to disastrous public health consequences (51).

## Conclusions

Rapid development of diagnostic tools and immune-based assays are important early interventions against the ongoing SARS-CoV-2 pandemic. The availability of serological assays that target a diverse range of viral antigen has no doubt assisted in the accurate diagnosis of COVID-19 patients. Essentially, data generated through serological studies can greatly aid in supplementing the results from qRT-PCR, as well as contribute to seroepidemiology, which has been shown to help in the design of virus elimination programs (52). Moving forward, this extensive collation of the current immunoassays against SARS-CoV-2 will provide insights toward monoclonal antibodies discovery and characterization for the development of a SARS-CoV-2 vaccine.

## Author Contributions

LN and LR conceived the presented idea. CL wrote the manuscript and prepared the figures. LN, LR, and RL revised the manuscript. All authors approved this manuscript for publication.

## Conflict of Interest

The authors declare that the research was conducted in the absence of any commercial or financial relationships that could be construed as a potential conflict of interest.
